# Identification of oxidative stress-related genes differentially expressed in Alzheimer’s disease and construction of a hub gene-based diagnostic model

**DOI:** 10.1038/s41598-023-34021-1

**Published:** 2023-04-26

**Authors:** Yanting Zhang, Hisanori Kiryu

**Affiliations:** Department of Computational Biology and Medical Sciences, Graduate School of Frontier Sciences, The University of Tokyo, 7-3-1 Hongo, Bunkyo-ku, Tokyo, 113-0033 Japan

**Keywords:** Computational biology and bioinformatics, Genetics

## Abstract

Alzheimer’s disease (AD) is the most prevalent dementia disorder globally, and there are still no effective interventions for slowing or stopping the underlying pathogenic mechanisms. There is strong evidence implicating neural oxidative stress (OS) and ensuing neuroinflammation in the progressive neurodegeneration observed in the AD brain both during and prior to symptom emergence. Thus, OS-related biomarkers may be valuable for prognosis and provide clues to therapeutic targets during the early presymptomatic phase. In the current study, we gathered brain RNA-seq data of AD patients and matched controls from the Gene Expression Omnibus (GEO) to identify differentially expressed OS-related genes (OSRGs). These OSRGs were analyzed for cellular functions using the Gene Ontology (GO) database and used to construct a weighted gene co-expression network (WGCN) and protein-protein interaction (PPI) network. Receiver operating characteristic (ROC) curves were then constructed to identify network hub genes. A diagnostic model was established based on these hub genes using Least Absolute Shrinkage and Selection Operator (LASSO) and ROC analyses. Immune-related functions were examined by assessing correlations between hub gene expression and immune cell brain infiltration scores. Further, target drugs were predicted using the Drug-Gene Interaction database, while regulatory miRNAs and transcription factors were predicted using miRNet. In total, 156 candidate genes were identified among 11046 differentially expressed genes, 7098 genes in WGCN modules, and 446 OSRGs, and 5 hub genes (MAPK9, FOXO1, BCL2, ETS1, and SP1) were identified by ROC curve analyses. These hub genes were enriched in GO annotations “Alzheimer’s disease pathway,” “Parkinson’s Disease,” “Ribosome,” and “Chronic myeloid leukemia.” In addition, 78 drugs were predicted to target FOXO1, SP1, MAPK9, and BCL2, including fluorouracil, cyclophosphamide, and epirubicin. A hub gene-miRNA regulatory network with 43 miRNAs and hub gene-transcription factor (TF) network with 36 TFs were also generated. These hub genes may serve as biomarkers for AD diagnosis and provide clues to novel potential treatment targets.

## Introduction

First described in 1906 by German psychiatrist Alois Alzheimer, Alzheimer’s disease (AD) is a progressive neurodegenerative disorder of the central nervous system that results in the gradual loss of memory and other cognitive capacities. The hallmarks of AD are the accumulation of extracellular protein plaques containing amyloid and intracellular neurofibrillary tangles (NTs) containing hyperphosphorylated tau protein. However, it is still uncertain if these plaques and NTs actually cause the associated neuronal and functional impairments. Moreover, epidemiological, neuroimaging, and neuropathological studies suggest that AD results from a combination of genetic, lifestyle, and environmental triggers and that multiple pathomechanisms contribute to neurodegeneration. Alzheimer’s disease is the most prominent cause of dementia^1^ and the sixth most prevalent cause of premature death worldwide. Over 55 million people globally currently suffer from dementia, and this number is expected to rise above 150 million by 2050 due to population aging. Further, more than 75$\%$ of these patients are undiagnosed and lack access to treatment and care^2^. Only four drugs have been approved for AD therapy, and these only treat symptoms to improve patient quality of life, but do not stop the progression of the underlying neurodegenerative processes^3^. Moreover, drug development for AD has a high failure rate, presumably due to the many unknown aspects of disease pathogenesis. Therefore, there is an urgent need to identify novel pathogenic mechanisms and therapeutic targets^4^. Mounting evidence suggests that normal age-related functional decline and diseases of aging arise at least in part from the accumulation of molecular damage due to oxidative stress and inflammation (^5,6^ for reviews). The oxidative stress (OS) theory of aging proposes that the slow and steady accumulation of macromolecular damage from reactive oxygen species (ROS) eventually leads to the functional impairment or irreversible loss of terminally differentiated cells^7^. Related theories include the mitochondrial theory of aging^8^, the cellular senescence theory of aging^9^, and the molecular inflammation theory of aging^10^. In all these theories, the accumulation of ROS-induced damage is posited as a potential mechanism leading to age-related functional decline, failure of endogenous repair mechanisms, and neurodegenerative diseases, of which AD is the most common^11–13^. It has been demonstrated that products of protein, DNA, RNA, and lipid oxidation (such as carbonyls, 3-nitrotyrosine, 8-oxo-7,8-dihydroguanine, and lipid peroxides) are substantially elevated in the brains of AD patients^14–17^. Moreover, oxidative damage to macromolecules is among the earliest signs of AD onset and an index of disease progression. In this study, we identified multiple OS-associated biomarkers in AD for the first time by comparing brain tissue gene expression profiles between patients and matched controls from the Gene Expression Omnibus (GEO), investigated the functional annotations of differentially expressed genes (DEGs), and constructed gene co-expression and protein-protein interaction (PPI) networks to define hub genes. We then constructed a diagnostic model based on these hub genes and used the miRNET database to predict hub gene targeted drugs and miRNAs as novel potential treatments.

## Results

### Identification of DEGs and module genes

**Figure 1:**
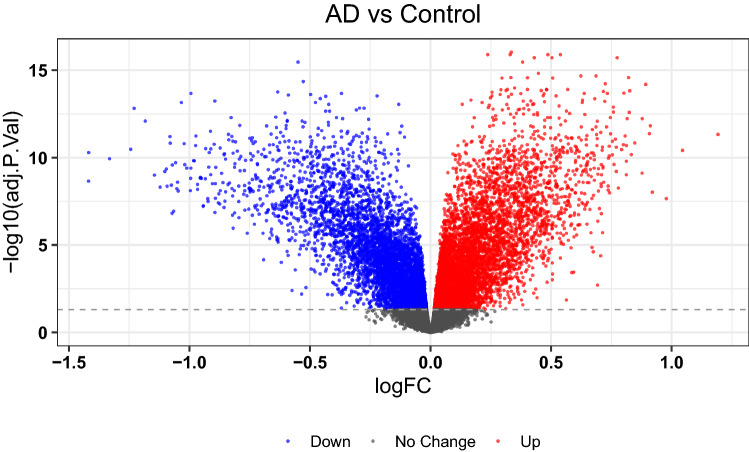
Volcano plot of differential gene expression between AD patients and matched controls from the GEO database.

**Figure 2:**
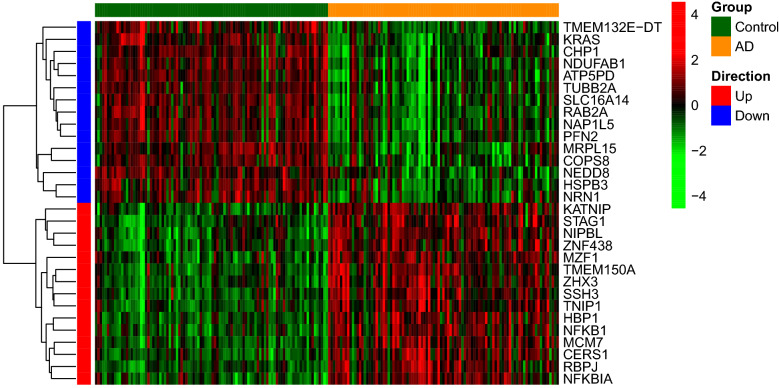
Heatmap of differentially expressed genes (DEGs) between AD patients and controls from the GEO database.

A total of 11046 differentially expressed genes (DEGs) were found between AD and control samples from the GEO GSE132903 dataset, of which 5805 were upregulated and 5241 downregulated (Fig. 1). The top 15 most significantly upregulated DEGs were TMEM132E-DT, KRAS, CHP1, NDUFAB1, ATP5PD, TUBB2A, SLC16A14, RAB2A, NAP1L5, PFN2, MRPL15, COPS8, NEDD8, HSPB3 and NRN1, and the top 15 most downregulated DEGs were KATNIP, STAG1, NIPBL, ZNF438, MZF1, TMEM150A, ZHX3, SSH3, TNIP1, HBP1, NFKB1, MCM7, CERS1, RBPJ and NFKBIA (Fig. 2).

**Figure 3:**
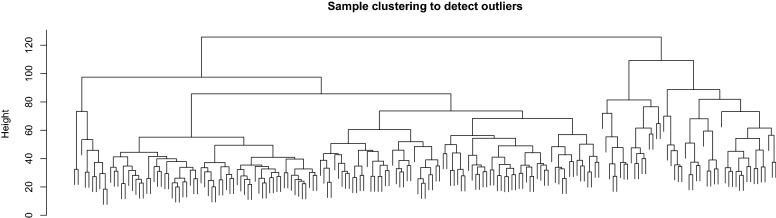
Outlier sample detection. The gene sets from all samples are contained in the dendrogram.

**Figure 4:**
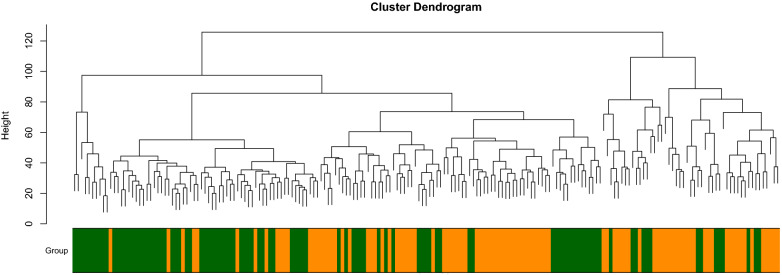
Clustering tree.

**Figure 5:**
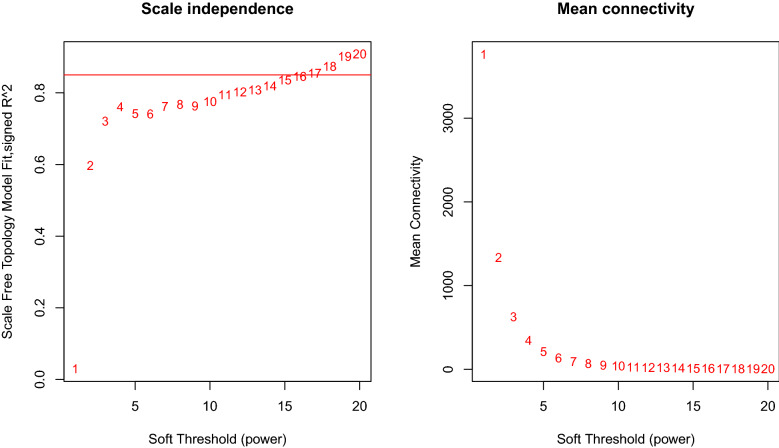
Soft threshold screening.

**Figure 6:**
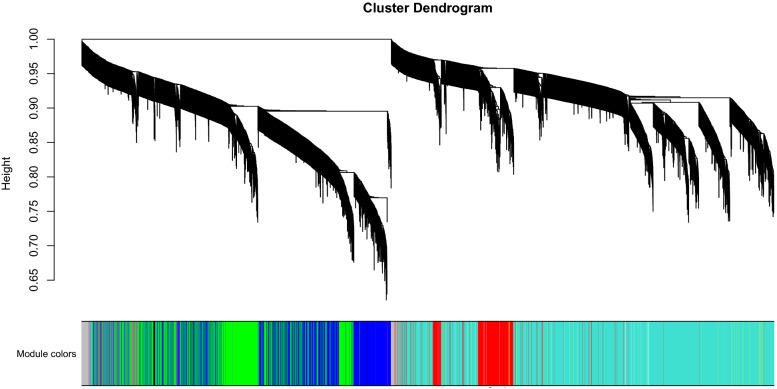
WGCNA modules.

**Figure 7:**
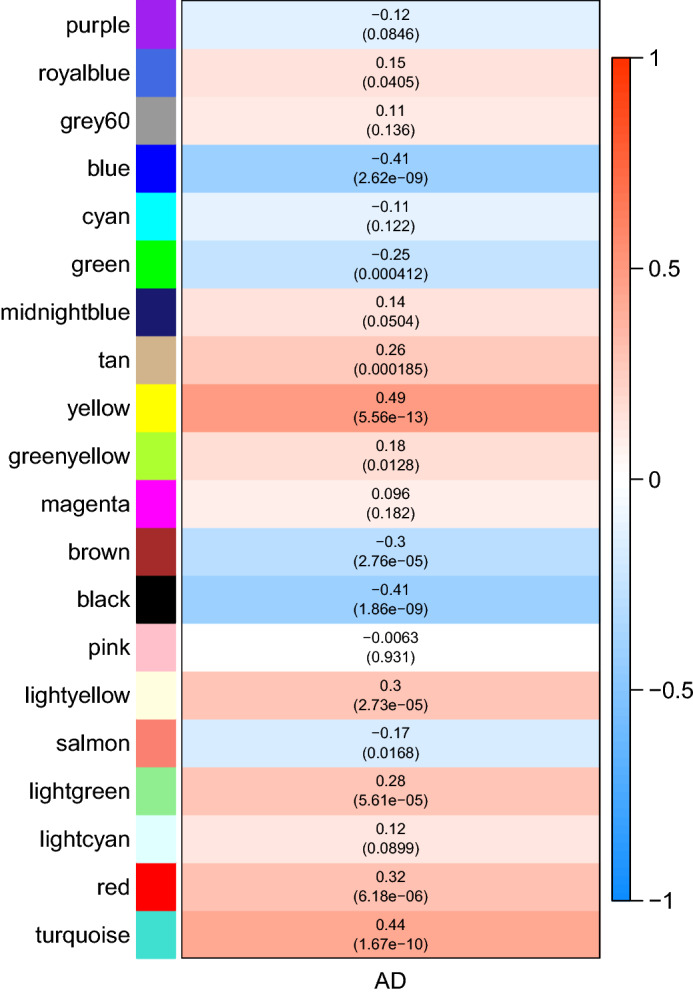
Module-disease correlations.

In weighted gene co-expression network cluster analysis, the gene sets of all samples were included in the dendrogram (i.e., there were no outliers) (Figs. 3,  4). The soft threshold was set at 17 (R2 $=$ 0.85) to construct a scale-free network (Fig. 5), and 20 modules were identified (Fig. 6). Based on the criteria (|Cor| > 0.4), blue, turquoise, yellow, and black modules, collectively including 7098 genes, were selected for further analysis (Fig. 7).

### Screening and functional enrichment of candidate genes

**Figure 8:**
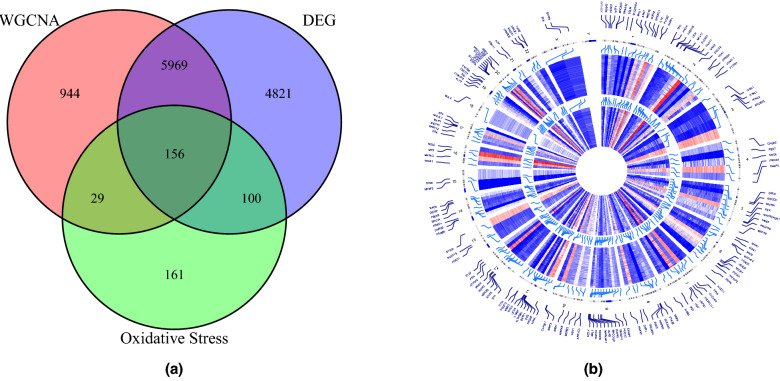
(**a**) Wayne diagram of candidate hub gene screening. (**b**) Chromosomal locations of candidate hub genes. The gene expression petterns of these genes in the GSE132903 datasets are represented in the inner circular heatmaps. Red indicates gene up-regulation, blue represents downregulation, and the outer circle of heatmap represents the control group, the inner circle of heatmap represents the disease group. The outer circle represents chromosomes; lines coming from each gene point to their specific chromosomal locations.

Analysis of the overlap among the 11046 DEGs, 7098 module genes, and 446 OSRGs yielded 156 candidate hub genes associated with OS and AD (Fig. 8a). These candidate hub genes were enriched in 1577 GO terms (1453 BP, 43 CC, 81 MF), including biological process (BP) terms “response to oxidative stress,” “cellular response to oxidative stress”, “cellular response to chemical stress”, and “response to reactive oxygen species,” cellular component (CC) terms “membrane raft”, “membrane microdomain”, and “mitochondrial matrix” (Fig. 10a), and molecular function (MF) terms “antioxidant activity”, “ubiquitin-like protein ligase binding”, and “ubiquitin protein ligase binding” (See Table 1 in the Supplementary Material). Additionally, candidate genes were enriched in 237 KEGG pathways, including “FOXO signaling pathway”, “MicroRNAs in cancer”, “AGE-RAGE signaling pathway in diabetic complications”, and “Shigellosis” (Fig. 10b and see Table 2 in the Supplementary Material).

**Figure 9:**
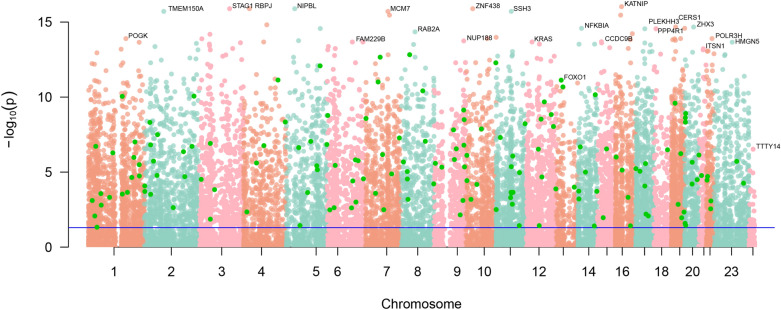
Manhattan plot of candidate genes.

**Figure 10:**
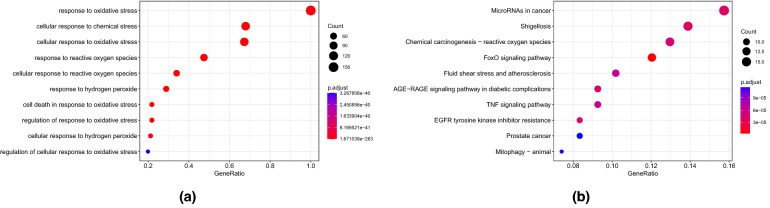
(**a**) GO enrichment analysis of candidate hub genes. (**b**) KEGG pathway enrichment analysis of candidate hub genes.

With two types of AD, early-onset and late-onset, both associated with autosomal dominant or sporadic inheritance^18^, studying chromosomal location can help researchers gain a deeper insight into genetic factors. The visualization of gene expression patterns and chromosomal locations of candidate hub genes in AD and normal controls were exhibited in Figs. 8b and 9, suggesting that these genes were distributed in all chromosomes except for chromosome Y, and there were most genes in chromosome 1.

### Identification of hub genes by co-expression analysis

**Figure 11:**
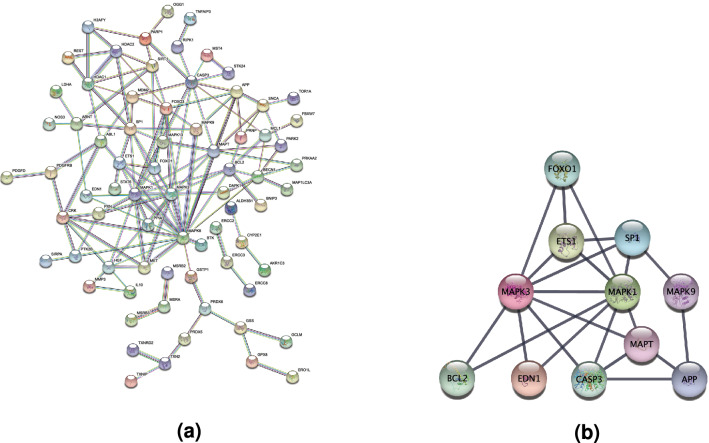
(**a**) Protein–protein interaction network constructed from candidate hub genes. (**b**) Hub sub-networks.

To identify hub genes (those most strongly interacting with the greatest number of other DEGs), a PPI network was constructed using the STRING website (Fig. 11a) and analyzed using Cytoscape, which yielded a sub-network with 11 candidates (MAPK3 (on chromosome 16), MAPK1 (on chromosome 22), SP1 (on chromosome 12), CASP3 (on chromosome 4), MAPT (on chromosome 17), BCL2 (on chromosome 18), APP (on chromosome 21), FOXO1 (on chromosome 13), ETS1 (on chromosome 11), MAPK9 (on chromosome 5), and EDN1 (on chromosome 6)) (Figs. 8b,  11b). We further validated these hub genes with screening thresholds p. adj < 0.01 and abs(logFC) > 0.585^19^, and the results demonstrate the reliability of these hub genes in terms of statistical significance (See Table 6 in the Supplementary Material). Of these, MAPK3 and MAPK1 were the most strongly connected to the rest of the network with connectivity scores of 15, followed by SP1 (10) and CASP3 (10) (See Fig. S1 in the Supplementary Material).

**Figure 12:**
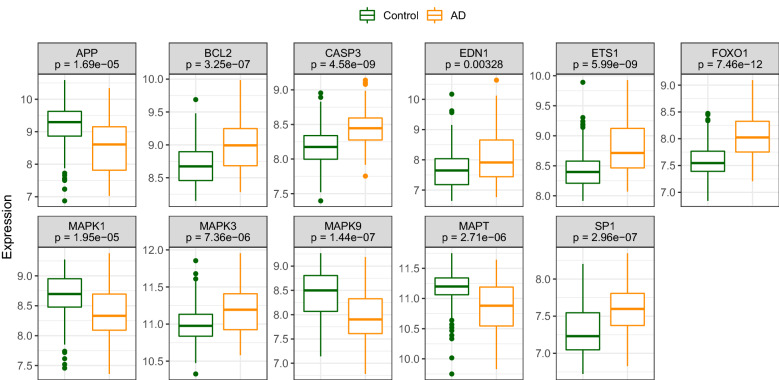
Expression levels of candidate hub genes in patients and matched controls.

**Figure 13:**
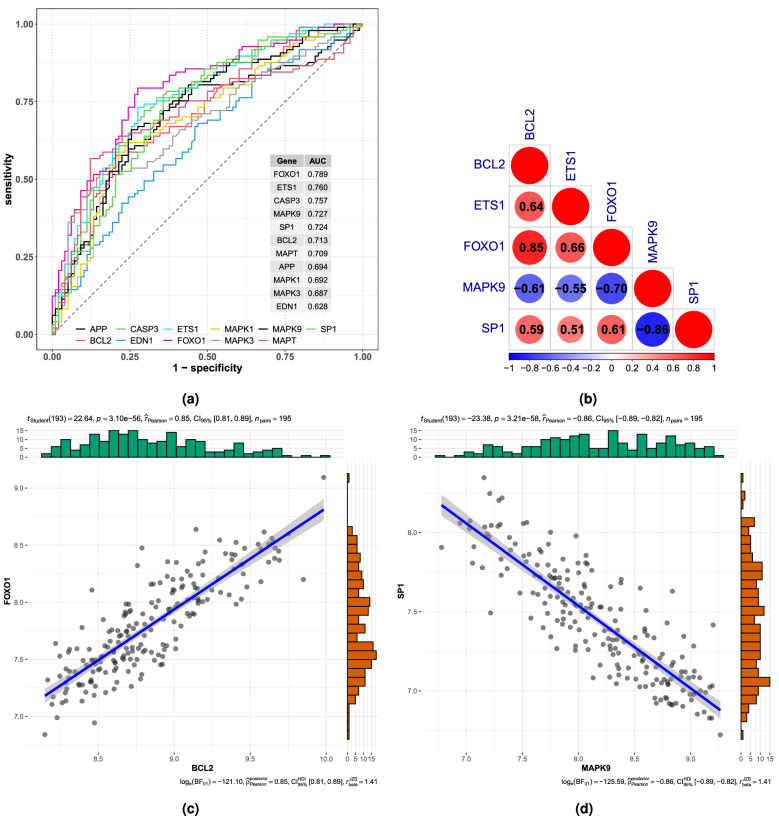
(**a**) ROC curve analysis of candidate hub genes. The seven with best performance for distinguishing AD from control samples according to area under the curve (AUC) were selected for further analyses. (**b**) Correlations between expression levels of various candidate hub gene pairs. (**c**) Correlation between FOXO1 and BCL2 expression. (**d**) Correlation between SP1 and MAPK9 expression.

All 11 candidate hub genes were expressed at different levels in AD and normal samples of the GSE132903 dataset, with APP, MAPK1, MAPK9, and MAPT expression levels higher in control samples and the rest higher in AD samples (Fig. 12). The capacity of each hub gene to distinguish AD from control samples according to expression level was then examined by ROC curve analysis, and the seven candidates with best discrimination performance (area under the ROC curve > 0.7), namely SP1, CASP3, MAPT, BCL2, FOXO1, ETS1, and MAPK9, were selected for further analyses (Fig. 13a).

Of these seven candidate hub genes in the GSE118553 dataset, all except MAPT were significantly differentially expressed between AD and normal samples, with CASP3 and MAPK9 expressed at higher levels in normal samples and SP1, BCL2, FOXO1, and ETS1 expressed at higher levels in the AD samples (see Fig. S5). The AUCs of the ROC curves for SP1, CASP3, BCL2, and MAPK9 were greater than 0.7 (see Fig. S6 in the supplementary material). Moreover, SP1, BCL2, FOXO1, ETS1, and MAPK9 were differentially expressed between AD and control samples from the GSE5281 dataset (see Fig. S7 in the Supplementary Material). Also, the AUCs of the ROC curves for SP1, BCL2, FOXO1, ETS1, and MAPK9 were greater than 0.7 (See Fig. S8 in the Supplementary Material). Based on these analyses, MAPK9, FOXO1, BCL2, ETS1, and SP1 were identified as hub genes. Co-expression analysis revealed that BCL2 and FOXO1 exhibited the strongest positive correlation, while MAPK9 and SP1 demonstrated the strongest negative correlation (Fig. 13b–d).

### Correlations between immune cell infiltration scores and hub gene expression levels

**Figure 14:**
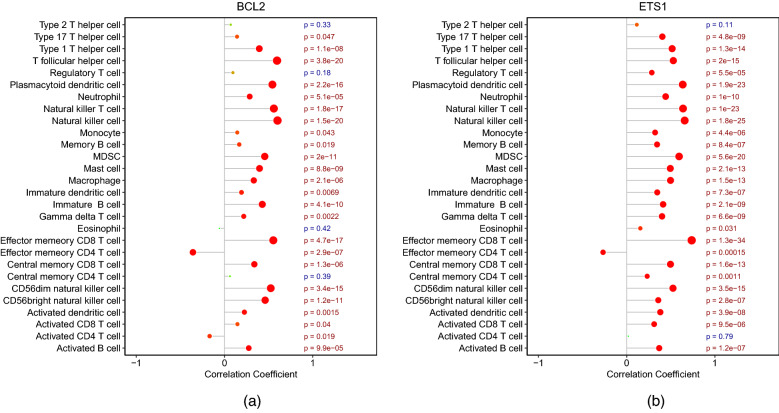
Correlations of immune cell infiltration scores with expression levels of BCL2 and ETS1.

To examine the effects of hub gene expression on neuroinflammation, we assessed associations with the brain infiltration of specific immune cell types. Expression of BCL2 was significantly correlated with the infiltration scores of 24 immune cell types (all considered except for Type 2 T helper cells, regulatory T cells, eosinophils, and central memory CD4 T cells). The correlations were positive with T follicular helper cell, natural killer cell, natural killer T cell, and effector memory CD8 T cell infiltration scores, and negative with effector memory CD4 T cell and Activated CD4 T cell infiltration scores (Fig. 14a). There was no significant correlation between ETS1 expression and either Type 2 T helper cell or activated CD4 T cell infiltration scores. ETS1 expression was negatively correlated only with effector memory CD4 T cell infiltration score, while all other correlations were positive (Fig. 14b). The expression levels of BCL2, ETS1, FOXO1, and SP1 were positively correlated with infiltration scores for the majority of immune cells (See Figs. S2, S3 in the Supplementary Material). In contrast, MAPK9 expression was positively correlated only with Type 2 T helper cell, regulatory T cell, effector memory CD4 T cell, central memory CD4 T cell, and activated CD4 T cell infiltration scores, but negatively correlated with the infiltration scores of most other immune cells (See Fig. S4 in the Supplementary Material).

### Gene set enrichment analysis

**Figure 15:**
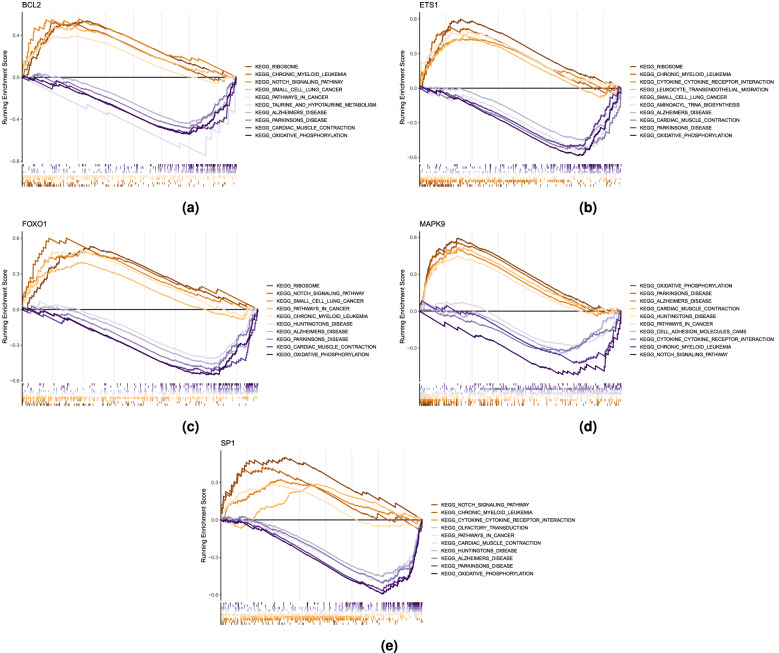
Gene set enrichment analysis for (**a**) BCL2, (**b**) ETS1, (**c**) FOXO1, (**d**) MAPK9, and (**e**) SP1.

Gene set enrichment analysis (GSEA) was then conducted to explore the molecular functions of these hub genes. All five hub genes are included in the “Alzheimer’s disease pathway’ (i.e., the Alzheimer’s disease pathway is overrepresented in the hub gene list), with MAPK9 downregulated and the remaining four upregulated. However, all five genes are also included in the list of genes implicated in “Parkinson’s disease’ and high expression levels of BCL2, ETS1, and FOXO1 are associated with “ribosome,” “chronic myeloid leukemia,” and “small cell lung cancer’ (Fig.  15a–e).

### Drugs, genes, and transcription factors regulating hub genes

**Figure 16:**
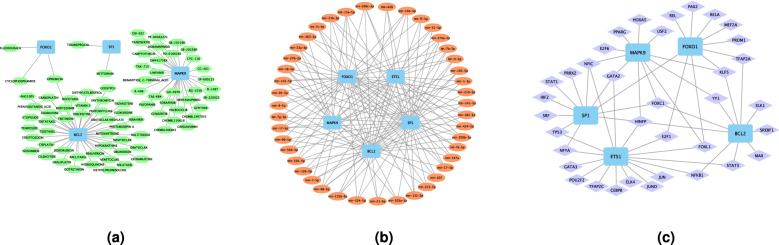
(**a**) Small-molecule drugs influencing hub gene expression or function. (**b**) MicroRNAs targeting hub genes. (**c**) Transcription factors regulating hub gene expression.

78 target drugs were predicted by FOXO1, SP1, MAPK9 and BCL2 (Fig. 16a and see Table 3 in the Supplementary material). For instance, there were 3 target drugs (Fluorouracil, Cyclophosphamide, epirubicin) of FOXO1, and 2 target drugs (Definite, Definite) of SP1. There were 21 drugs predicted by MAPK9, such as Camptothecin, Chembl406845, OSI-632, PD-0166285 and SB-220025, and 43 drugs were predicted by BCL2, including Hydroquinone, Pentoxifylline, Edaravone, Gossypol, etc.

The Drug-Gene Interaction database identified 78 small-molecule drugs potentially influencing hub gene expression or function. Moreover, a hub gene-miRNA regulatory network and a hub gene-TF network generated using the miRNet database identified 43 targeted miRNAs (Fig. 16b and see Table 4 in the Supplementary Material) and 36 TFs. For example, GATA2 and NFIC are TFs controlling SP1, ETS1, and MAPK9 expression (Fig. 16c and Table 5 in the Supplementary Material).

### Diagnostic model based on 5 hub genes

**Figure 17:**
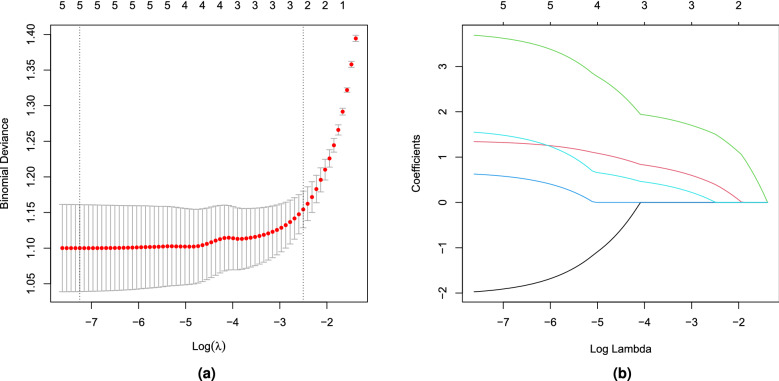
LASSO regression model $\lambda$-screening.

**Figure 18:**
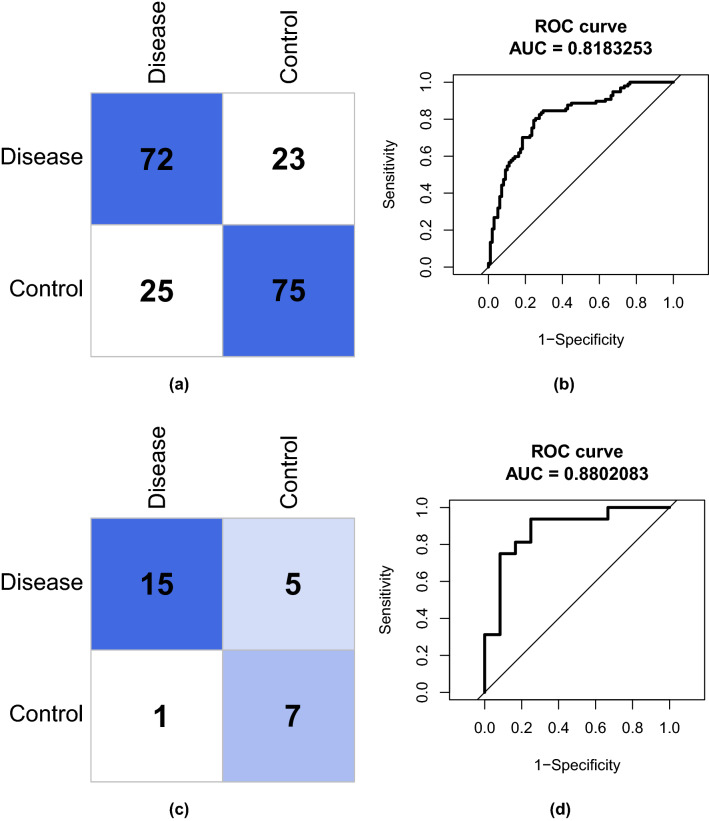
(**a**) Confusion matrix for training set. (**b**) ROC curve for training set. (**c**) Confusion matrix for validation set. (**d**) ROC curve for validation set.

A diagnostic model was then constructed based on expression levels of the five hub genes using LASSO regression and ROC analyses. For the optimal model developed using the training dataset, lambda.min = 0.00071235 (Fig. 17), and the coefficients of the five genes were BCL2 $= -1.9400531$, ETS1 $=$ 1.3313010, FOXO1 $=$ 3.6540838, MAPK9 $=$ 0.6012298, and SP1 $=$ 1.5144388. Moreover, the confusion matrix showed that the specificity of the model reached 0.74, accuracy 0.75, and recall 0.76 for the training set (Fig. 18a). Further, the AUC of the ROC curve was 0.818, indicating good discrimination performance (Fig. 18b).

Model performance was then validated using the GEO dataset GSE5281 (Fig. 18c). The confusion matrix showed that the model achieved similar precision (0.94), accuracy (0.786), and recall (0.75) on this dataset. The AUC of the ROC curve was also similar (0.88), underscoring the general utility of the diagnostic model (Fig. 18d).

## Discussion

A prominent early feature of AD is the accumulation of OS markers^20–22^, which results from an imbalance between endogenous ROS generation and cellular antioxidant capacity. The primary endogenous sources of ROS, such as mitochondrial respiration and various enzymatic reactions, and the physiological mechanisms regulating endogenous antioxidants are well described. Moreover, many exogenous factors are known to directly scavenge ROS and/or enhance endogenous antioxidant capacity. Therefore, limiting OS and associated damage may be a particularly promising area of AD drug research and development^4^. Here we identified numerous potential OS markers by comparing gene expression profiles between patients and matched controls. X chromosome aberrations have been confirmed by previous studies^23^ to be highly associated with oxidative stress and could be a breakthrough for future disease-related investigations. Meanwhile, mutations in three different genes on chromosomes 1, 14, and 21 were reported to result in autosomal dominant forms of familial Alzheimer’s disease, that is^24^, the APP gene on chromosome 21, the PS1 gene on chromosome 14, and the PS2 gene onchromosome 1^23,25^. Based on the visualization of the chromosomal location of candidate hub genes (Figs. 8b and 9), it was showed that the AD-related differential genes identified were distributed on these crutial chromosomes, with the most differential candidates on chromosome 1. Meanwhile, chromosome 6 locus and chromosome 12 are highly associated with late-onset Alzheimer’s disease^26^, while AD is also highly neuropathologically similar to familial British dementia and familial Danish dementia, which are highly associated with chromosome 13^27^. It further indicates that these chromosomes are significantly associated with AD development.

Then we defined five major hub genes of a network associated with oxidative stress in AD patients. These detected hub genes included the stress-associated signaling factor MAPK9, the negative regulator of apoptosis BCL2, and three transcription factors, FOXO1, ETS1, and SP1, associated with various stress and protective responses. A diagnostic model based on the expression levels of these five genes distinguished patients from controls with high sensitivity and selectivity.

Expression of MAPK9 was consistently higher in normal samples while the expression levels of FOXO1, BCL2, ETS1, and SP1 were higher in AD samples (See Fig. S5 in the Supplementary Material). Construction of hub gene-miRNA and hub gene-TF networks identified multiple regulatory pathways contributing to these differences in expression, and many of the interactions of DEGs with TFs and miRNAs we identified were demonstrated experimentally in past studies^28–32^. These DEGs and upstream TFs are involved in a variety of biological processes, such as the immune response, apoptosis, and vascular morphogenesis. Previous studies have strongly implicated BCL2 and FOXO1 in neuronal survival, while FOXO and MAPK signaling pathways are known as important regulators of the cell cycle and autophagy/apoptosis^33^. Previous studies have also reported BCL2 overexpression in the fontal cortex of AD patients and suggested that this reflects a compensatory anti-apoptotic response that is stronger in frontal cortex than in other brain regions^34–36^. There is also compelling evidence for a relationship between dysregulated BCL2 expression and impaired learning and memory. High FOXO1 expression may be linked to age-related neurodegeneration through regulation of advanced glycation end product (AGE) signaling pathways, cellular senescence mechanisms, autophagic pathways, and apoptotic pathways induced by accumulation of misfolded proteins or cellular damage^37,38^. Elevated ETS1 expression may be associated with microglia activation and proliferation, a critical initiating event in neuroinflammation^39,40^. The transcription factor SP1 is strongly implicated in the regulation of OS response genes, and notably also in the expression of amyloid precursor protein (APP) and tau protein. Moreover, SP1 has been found to be significantly upregulated in the frontal cortex of AD patients^30,41,41,42^. Finally, MAPK9 (also known as JNK2) plays an important role in the oxidative stress response^43^, and many studies have confirmed that JNK signaling is associated with aging^44,45^.

A diagnostic model constructed based on the expression levels of these five genes (MAPK9, BCL2, FOXO1, ETS1, and SP1) using LASSO regression and ROC analysis demonstrated high accuracy for distinguishing patients from matched controls in both training and validation datasets. Therefore, this model may have clinical value for the early diagnosis of AD. BCL2 is a primary negative regulator of apoptosis that may be induced in response to stressors such as DNA damage. In contrast, however, neurons with neurofibrillary tangles (NTs), a hallmark of AD, exhibit downregulated BCL2 expression and thus potentially greater susceptibility to apoptosis^46^. Recent research has confirmed that BCL2 also regulates intracellular Ca$^2+$ signaling by influencing mitochondrial- and endoplasmic reticulum-based mechanisms for Ca2 homeostasis, and aberrant neuronal Ca$^2+$ signaling has long been implicated in AD as well as other neurodegenerative disorders. Therefore, anti-apoptotic BCL2 and its derived peptides may have potential for therapeutic prevention of AD pathogenesis^47^. ETS1 is a pro-angiogenic transcription factor, and enhanced intravascular immunoreactivity as well as extravasation have been documented in the cortical microvasculature of AD brain. The expression of ETS1 is also strongly correlated with expression of tumor necrosis factor-α (TNF-α), an important pro-inflammatory cytokine released by reactive microglia (ref). Therefore the expression pattern of ETS1 in the brain of AD patients is likely of great importance to disease progression^39^. The FOXO transcription factors regulate the balance between cell longevity and tumor suppression by controlling the expression of proteins involved in cell cycle arrest, stress resistance, and apoptosis. Therefore, among these downstream proteins are numerous potential molecular targets for the treatment of AD^48^. The c-Jun N-terminal kinase (JNK) signaling cascade is triggered in response to a broad spectrum of stressors^49^. Of particular relevance to AD, JNK-MAPK proteins are involved in the regulation of apoptosis during oxidative damage through phosphorylation of effector proteins or transcription factors, which may lead to dysregulation of tau phosphorylation and accumulation of hyperphosphorylated tau, the major component of NTs^50,51^. The transcription factor SP1 also responds to inflammatory signals, such as those induced by neuronal damage. Elevated expression of SP1 in AD samples may be a protective response, so it would be intriguing to investigate whether regulation of SP1 by drugs such as mithramycin can improve AD symptoms such as memory dysfunction^52^. The known functions of these hub genes are also consistent with the strong correlations with immune infiltration scores and enrichment analyses using GO and DEGG databases. The identification of these hub genes may thus facilitate the development of novel disease-modifying therapies as well as symptom-targeted drugs.

In this study, we firstly identified five hub genes that target oxidative stress (OS) process in Alzheimer’s disease (AD) through bioinformatic analysis, which were then examined for biological processes and pathways, associations with brain infiltration of specific immune cells, and for their capacity as a diagnostic model to distinguish patients from controls. While further experimental validation is required to verify OS- and AD-related functions, the high performance of the diagnostic model and previous investigations strongly implicate these five hub genes, their downstream targets for transcriptional regulation, and upstream regulators (including numerous miRNAs) in the OS response in AD. It is worth that 78 drugs identified that target these hub genes have been not proven to mitigate AD, and no EST1-modulating drugs were detected, suggesting that testing of these agents in AD animal models and development of small-molecule EST1 regulators are potentially productive areas for future research. The distribution of candidate hub genes on chromosomes may provide insights for future studies on potential pathogenic mechanisms underlying potential pathogenesis on other chromosomes. Also, it is vital to supplement this model to distinguish AD from Parkinson’s disease.

## Methods

### Data sources

Datasets GSE132903, GSE118553, and GSE5281 were obtained from the National Center for Biotechnology Information database (https://www.ncbi.nlm.nih.gov/). The GSE132903 dataset contains expression profiles of middle temporal gyrus from 97 Alzheimer’s disease patients and 98 non-demented controls, GSE118553 includes transcriptomic data of brain tissues from 52 AD patients and 31 controls, and GSE5281 comprises gene expression data of brain samples from 16 AD patients and 11 controls. Moreover, 446 oxidative stress-related genes (OSRGs) were obtained from the Gene Ontology (GO) database (Data cohort: GO:0006979). The minimum age of study subjects in these datasets is 70 years.

### Identification and functional enrichment analysis of candidate genes influencing the oxidative stress response in Alzheimer’s disease

The Limma package (version 3.50.1) was used to perform differential expression analysis between AD and control samples of the GSE132903 dataset. In this study, since the training set is microarray data with overall small logFC values, the screening threshold of DEGs was adjusted p value (p. adj) < 0.05^53^. Considering that a large number of genes were available in the original dataset, the adjusted p-values provided a good balance between finding statistically significant genes and limiting the occurrence of false positives by implementing a Benjamini-Hochberg (BH) correction^54^ to control the false discovery rate of the entire significant genes, and considered for multiple testing. Further, the traits of AD patients and normal controls of the GSE132903 dataset were including in WGCNA analysis (WGCNA version 1.70-3)^55^ to define gene modules related to AD. Briefly, samples were first clustered to determine whether there were outliers that needed to be removed. Then, the optimal soft threshold was defined to ensure that gene interactions maximally conformed to a scale-free distribution. Based on the optimal soft threshold, a gene dendrogram was plotted using the dynamic tree cutting algorithm. After calculating the correlations between each module and traits, the modules with |Cor| > 0.4 were selected as key modules and the component genes were included as module genes for subsequent analyses. Candidate genes potentially influencing the OS response in AD were then identified by the overlap among DEGs, module genes, and OSRGs. The GO and KEGG enrichment analyses of candidate genes were performed using clusterProfiler (version 4.2.2), with a significance criterion of p < 0.05. Furthermore, genomic location distributions and expression levels of the candidate genes were visualized using OmicCircos (version 1.32.0).

### Identification and correlation analysis of hub genes

A PPI network of the hub candidate genes was constructed using the STRING website, and core network genes selected using the MCODE plug-in of Cytoscape (version 3.8.2). The genes in the core network were then evaluated for distinguishing AD from normal samples using ROC curve analysis, and those with AUC > 0.7 were selected. These candidate hub genes were further verified by ROC analysis using GSE118553 and GSE5281 dataset samples. Candidates showing reliable group classification performance in all datasets were selected as the final hub genes. Subsequently, the correlation in expression level between each hub gene pair was calculated and scatter plots drawn for the gene pairs with strongest positive and negative correlations.

### Immune infiltration analysis and GSEA

Neuroinflammation is a major driver of AD progression, so we examined if these hub genes were associated with the infiltration of various immune cells^56^. Correlations with the infiltration of 28 immune cell types in samples from GSE132903 were estimated by single-sample (ss)GSEA using GSVA (version 1.42.1). To further explore the functions of these hub genes, samples with high and low expression were analyzed using The Molecular Signatures Database (MSigDB). Among the significantly enriched KEGG pathways (p. adj < 0.05), the top five with normalized enrichment score (NES) > 0 and NES < 0 were selected for presentation.

### Regulatory networks and target drugs of hub genes

The miRNAs and transcription factors (TFs) regulating hub genes were predicted using the miRNET database^57^. To improve the clarity of network diagrams, only miRNAs targeting at least three hub genes were screened and visualized. Additionally, drugs influencing expression of hub genes were predicted using the Drug–Gene Interaction database (DGIdb)^58^, which includes drug–gene interaction data from 30 sources.

### Construction of a diagnostic model

A diagnostic model based on expression levels of the five hub genes was then established using GSE132903 as the training set. The expression levels of all hub genes were extracted and subjected to LASSO regression analysis to derive coefficients for diagnostic model construction. To evaluate the effectiveness of the diagnostic model, all samples in GSE132903 were categorized as disease or control, and the effectiveness of this categorization verified by plotting a confusion matrix and ROC curves. In addition, these analyses were repeated using GSE5281 as an external validation set.

## Supplementary Information

Supplementary Figures.

Supplementary Table 1.

Supplementary Table 2.

Supplementary Table 3.

Supplementary Table 4.

Supplementary Table 5.

Supplementary Table 6.

## Data Availability

The datasets generated and/or analyzed during the current study are available in the GEO repository. It is a public free repository database and the links for these datasets are provided below, GSE132903: https://www.ncbi.nlm.nih.gov/geo/query/acc.cgi?acc=GSE132903. GSE5281: https://www.ncbi.nlm.nih.gov/geo/query/acc.cgi?acc=GSE5281. GSE118553: https://www.ncbi.nlm.nih.gov/geo/query/acc.cgi?acc=GSE118553.

## References

[1] Qiu C, Kivipelto M, von Strauss E (2009). Epidemiology of Alzheimer’s disease: Occurrence, determinants, and strategies toward intervention. Dialogues Clin. Neurosci..

[2] Gauthier, S., Rosa-Neto, P., Morais, J.A. & Webster, C. *World Alzheimer Report 2021: Journey Through the Diagnosis of Dementia*. https://www.alzint.org/resource/world-alzheimer-report-2021/ (2021).

[3] Chen X, Pan W (2014). The treatment strategies for neurodegenerative diseases by integrative medicine. Integr. Med. Int..

[4] Blaikie L, Kay G, Kong Thoo Lin P (2019). Current and emerging therapeutic targets of Alzheimer’s disease for the design of multi-target directed ligands. MedChemComm.

[5] Liguori I, Russo G, Curcio F, Bulli G, Aran L, Della-Morte D, Gargiulo G, Testa G, Cacciatore F, Bonaduce D, Abete P (2018). Oxidative stress, aging, and diseases. Clin. Interv. Aging.

[6] Kregel KC, Zhang HJ (2007). An integrated view of oxidative stress in aging: Basic mechanisms, functional effects, and pathological considerations. Am. J. Physiol.-Regulat. Integr. Comp. Physiol..

[7] Hagen, T. M. Oxidative stress, redox imbalance, and the aging process. *Antioxid Redox Signal* **5**(5), 503–506. 10.1089/152308603770310149 (2003). 10.1089/152308603770310149 14580304

[8] Wallace DC (2005). A mitochondrial paradigm of metabolic and degenerative diseases, aging, and cancer: A dawn for evolutionary medicine. Annu. Rev. Genet..

[9] Beauséjour CM, Krtolica A, Galimi F, Narita M, Lowe SW, Yaswen P, Campisi J (2003). Reversal of human cellular senescence: roles of the p53 and p16 pathways. EMBO J..

[10] Chung HY, Sung B, Jung KJ, Zou Y, Yu BP (2006). The molecular inflammatory process in aging. Antioxid. Redox Signal.

[11] William R (1997). Markesbery oxidative stress hypothesis in Alzheimer’s disease. Free Radic. Biol. Med..

[12] Smith MA, Rottkamp CA, Nunomura A, Raina AK, Perry G (2000). Oxidative stress in Alzheimer’s disease. Biochim. Biophys. Acta (BBA)-Mol. Basis Dis..

[13] Zhao Y, Zhao B (2013). Oxidative stress and the pathogenesis of Alzheimer’s disease. Oxid. Med. Cell Longev..

[14] Beal MF (2002). Oxidatively modified proteins in aging and disease. Free Radic. Biol. Med..

[15] Head E (2008). Oxidative damage and cognitive dysfunction: Antioxidant treatments to promote healthy brain aging. Antioxid. Redox Signal.

[16] Praticó D, Sung S (2014). Lipid peroxidation and oxidative imbalance: early functional events in Alzheimer’s disease. Oxid. Med. Cell. Longev..

[17] Williams TI, Lynn BC, Markesbery WR, Lovell MA (2006). Increased levels of 4-hydroxynonenal and acrolein, neurotoxic markers of lipid peroxidation, in the brain in mild cognitive impairment and early Alzheimer’s disease. Neurobiol. Aging.

[18] Bekris LM, Yu CE, Bird TD, Tsuang DW (2010). Genetics of Alzheimer disease. J. Geriatr. Psychiatry Neurol..

[19] Gu X, Lai D, Liu S, Chen K, Zhang P, Chen B, Huang G, Cheng X, Lu C (2022). Hub genes, diagnostic model, and predicted drugs related to iron metabolism in Alzheimer’s disease. Front Aging Neurosci..

[20] Ansari MA, Scheff SW (2010). Oxidative stress in the progression of Alzheimer disease in the frontal cortex. J. Neuropathol. Exp. Neurol..

[21] Markesbery WR, Lovell MA (2006). DNA oxidation in Alzheimer’s disease. Antioxid. Redox Signal.

[22] Moreira PI, Nunomura A, Nakamura M, Takeda A, Shenk JC, Aliev G, Smith MA, Perry G (2008). Nucleic acid oxidation in Alzheimer disease. Free Radic. Biol. Med..

[23] Kumar M, Pathak D, Venkatesh S, Kriplani A, Ammini AC, Dada R (2012). Chromosomal abnormalities and oxidative stress in women with premature ovarian failure (POF). Indian J. Med. Res..

[24] Bird TD, Levy-Lahad E, Poorkaj P, Sharma V, Nemens E, Lahad A, Lampe TH, Schellenberg GD (1996). Wide range in age of onset for chromosome 1-related familial Alzheimer’s disease. Ann. Neurol..

[25] St George-Hyslop PH (2000). Molecular genetics of Alzheimer’s disease. Biol. Psychiatry.

[26] Naj Adam C (2010). Dementia revealed: Novel chromosome 6 locus for late-onset Alzheimer disease provides genetic evidence for folate-pathway abnormalities. PLOS Genet..

[27] Rostagno A (2002). Complement activation in chromosome 13 dementias. similarities with Alzheimer’s disease. J. Biol. Chem..

[28] Turk A, Kunej T, Peterlin (2021). MicroRNA-target interaction regulatory network in Alzheimer’s disease. J. Pers. Med..

[29] Wang X (2009). MIR-34a, a microRNA up-regulated in a double transgenic mouse model of Alzheimer’s disease, inhibits bcl2 translation. Brain Res. Bull..

[30] Villa C (2013). Expression of the transcription factor sp1 and its regulatory HSA-MIR-29b in peripheral blood mononuclear cells from patients with Alzheimer’s disease. J. Alzheimer’s Dis..

[31] Lau P (2013). Alteration of the microRNA network during the progression of Alzheimer’s disease. EMBO Mol. Med..

[32] Zhu X, Lee H-G, Raina AK, Perry G, Smith MA (2002). The role of mitogen-activated protein kinase pathways in Alzheimer’s disease. Neurosignals.

[33] Santos-Lobato BL, Vidal AF, Ribeiro-Dos-Santos Â (2021). Regulatory miRNA-mRNA networks in Parkinson’s disease. Cells.

[34] Bei R (2006). Immunity to extracellular matrix antigens is associated with ultrastructural alterations of the stroma and stratified epithelium basement membrane in the skin of hashimotos thyroiditis patients. Int. J. Immunopathol. Pharmacol..

[35] Niu Q, Yang Y, Zhang Qea (2007). The relationship between bcl-2 gene expression and learning & memory impairment in chronic aluminum-exposed rats. Neurotox Res..

[36] Midulla F (2006). Cytokines in the nasal washes of children with respiratory syncytial virus bronchiolitis. Int. J. Immunopathol. Pharmacol..

[37] Liu L, Bai J, Liu F, Xu Y, Zhao M, Zhao C, Zhou Z (2022). Cross-talking pathways of forkhead box o1 (foxo1) are involved in the pathogenesis of Alzheimer’s disease and Huntington’s disease. Oxid. Med. Cell Longev..

[38] Tan SH, Shui G, Zhou J, Shi Y, Huang J, Xia D, Wenk MR, Shen HM (2014). Critical role of scd1 in autophagy regulation via lipogenesis and lipid rafts-coupled akt-foxo1 signaling pathway. Autophagy.

[39] Jantaratnotai N, Ling A, Cheng J, Schwab C, McGeer PL, McLarnon JG (2013). Upregulation and expression patterns of the angiogenic transcription factor Ets-1 in Alzheimer’s disease brain. J Alzheimers Dis.

[40] Gjoneska E, Pfenning A, Mathys MH (2015). Conserved epigenomic signals in mice and humans reveal immune basis of Alzheimer’s disease. Nature.

[41] Citron BA, Dennis JS, Zeitlin RS, Echeverria V (2008). Transcription factor sp1 dysregulation in Alzheimer’s disease. J. Neurosci. Res..

[42] Santpere G, Nieto M, Puig B, Ferrer I (2006). Abnormal sp1 transcription factor expression in Alzheimer disease and tauopathies. Neurosci. Lett..

[43] Wang MC, Bohmann D, Jasper H (2003). JNK signaling confers tolerance to oxidative stress and extends lifespan in Drosophila. Dev. Cell.

[44] Oh SW, Mukhopadhyay A, Svrzikapa N, Jiang F, Davis RJ, Tissenbaum HA (2005). JNK regulates lifespan in *Caenorhabditis elegans* by modulating nuclear translocation of forkhead transcription factor/DAF-16. Proc. Natl. Acad. Sci. USA.

[45] Kim HJ, Jung KJ, Yu BP, Cho CG, Chung HY (2002). Influence of aging and calorie restriction on MAPKS activity in rat kidney. Exp. Gerontol..

[46] Su JH, Satou T, Anderson AJ, Cotman CW (1996). Up-regulation of Bcl-2 is associated with neuronal DNA damage in Alzheimer’s disease. Neuroreport.

[47] Callens Manon, Kraskovskaya Nina, Derevtsova Kristina, Annaert Wim, Bultynck Geert, Bezprozvanny Ilya, Vervliet Tim (2021). The role of Bcl-2 proteins in modulating neuronal Ca$$^2+$$ signaling in health and in Alzheimer’s disease. Biochim. Biophys. Acta (BBA)-Mol. Cell Res..

[48] Manolopoulos KN, Klotz LO, Korsten P, Bornstein SR, Barthel A (2010). Linking Alzheimer’s disease to insulin resistance: The foxo response to oxidative stress. Mol. Psychiatry.

[49] Gehi BR, Gadhave K, Uversky VN, Giri R (2022). Intrinsic disorder in proteins associated with oxidative stress-induced JNK signaling. Cell Mol. Life Sci..

[50] Sinha K, Das J, Pal PB, Sil PC (2013). Oxidative stress: The mitochondria-dependent and mitochondria-independent pathways of apoptosis. Arch. Toxicol..

[51] Zhang C, Tan Z, Xie Y, Zhao Y, Huang TY, Lu Z, Luo H, Can D, Xu H, Zhang YW, Zhang X (2019). Appoptosin mediates lesions induced by oxidative stress through the JNK–Foxo1 pathway. Front. Aging Neurosci..

[52] Citron, B. A., Saykally, J. N., Cao, C., Dennis, J. S., Runfeldt, M. & Arendash, G. W. Transcription factor sp1 inhibition, memory, and cytokines in a mouse model of Alzheimer’s disease. *Am. J. Neurodegener. Dis.* **4(2)**, 40–48 (2015). PMC4700125 26807343

[53] Udayaraja GKEI (2021). Network-based gene deletion analysis identifies candidate genes and molecular mechanism involved in clear cell renal cell carcinoma. J. Genet..

[54] Benjamini YHY (1995). Controlling the false discovery rate: A practical and powerful approach to multiple testing. J. R. Stat. Soc. Ser. B.

[55] Langfelder P, Horvath S (2008). WGCNA: An r package for weighted correlation network analysis. BMC Bioinform..

[56] Leng F, Edison P (2021). Neuroinflammation and microglial activation in Alzheimer disease: Where do we go from here?. Nat. Rev. Neurol..

[57] Chang L, Zhou G, Soufan O, Xia J (2020). miRNet 2.0: Network-based visual analytics for miRNA functional analysis and systems biology. Nucleic Acids Res..

[58] Cotto, K. C. *et al.* Dgidb 3.0: A redesign and expansion of the drug–gene interaction database. *Nucleic Acids Res.* **4**, 46. 10.1093/nar/gkx1143 (2018). 10.1093/nar/gkx1143 PMC5888642 29156001

