# Supplementary material for: Identification of oxidative stress-related genes differentially expressed in Alzheimer’s disease and construction of a hub gene-based diagnostic model
Source: Sci Rep. 2023 Apr 26;13:6817. doi: 10.1038/s41598-023-34021-1 (PMC10133299; doi:10.1038/s41598-023-34021-1)
Supplement: Supplementary file 1 — Supplementary Figures. [file 41598_2023_34021_MOESM1_ESM.pdf]

Identification of Oxidative Stress-related Genes Differentially Expressed in Alzheimer's Disease and Construction of a Hub Gene-based Diagnostic Model: supplemental document

Yanting Zhang[1,*] and Hisanori Kiryu[2]

[1,2]Department of Computational Biology and Medical Sciences, Graduate School of Frontier Sciences, The University of Tokyo, 7-3-1 Hongo, Bunkyo-ku, 113-0033, Tokyo, Japan
[*]yantingzhang0830@gmail.com

FIGURES

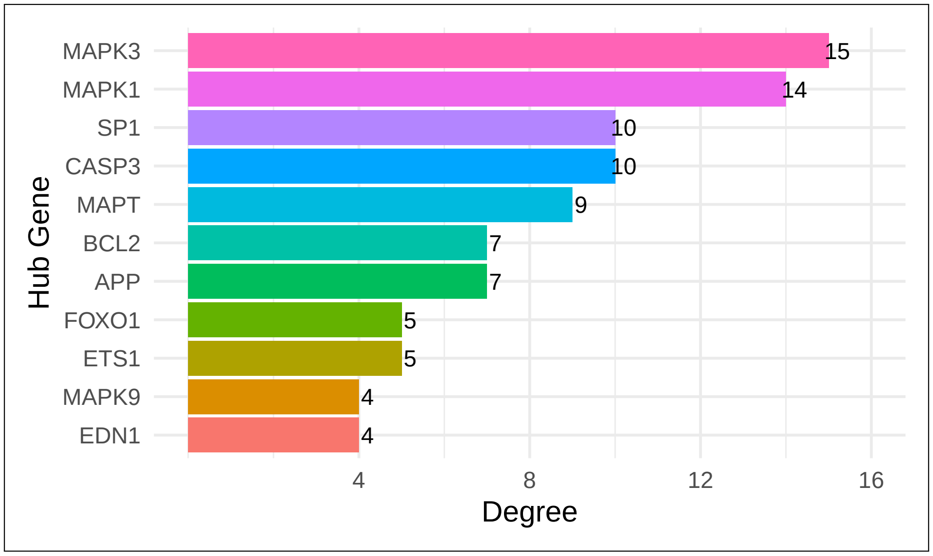

Fig. S1. Hub Gene Connectivity.

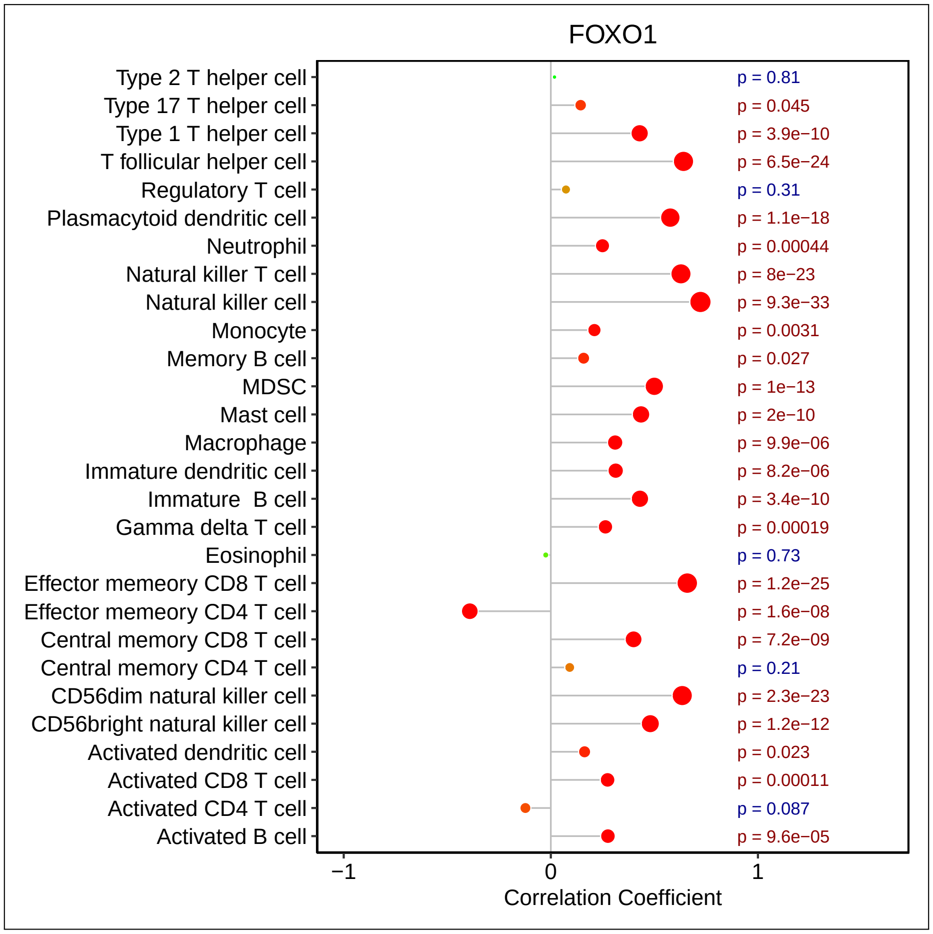

Fig. S2. Immune cell correlation (FOXO1).

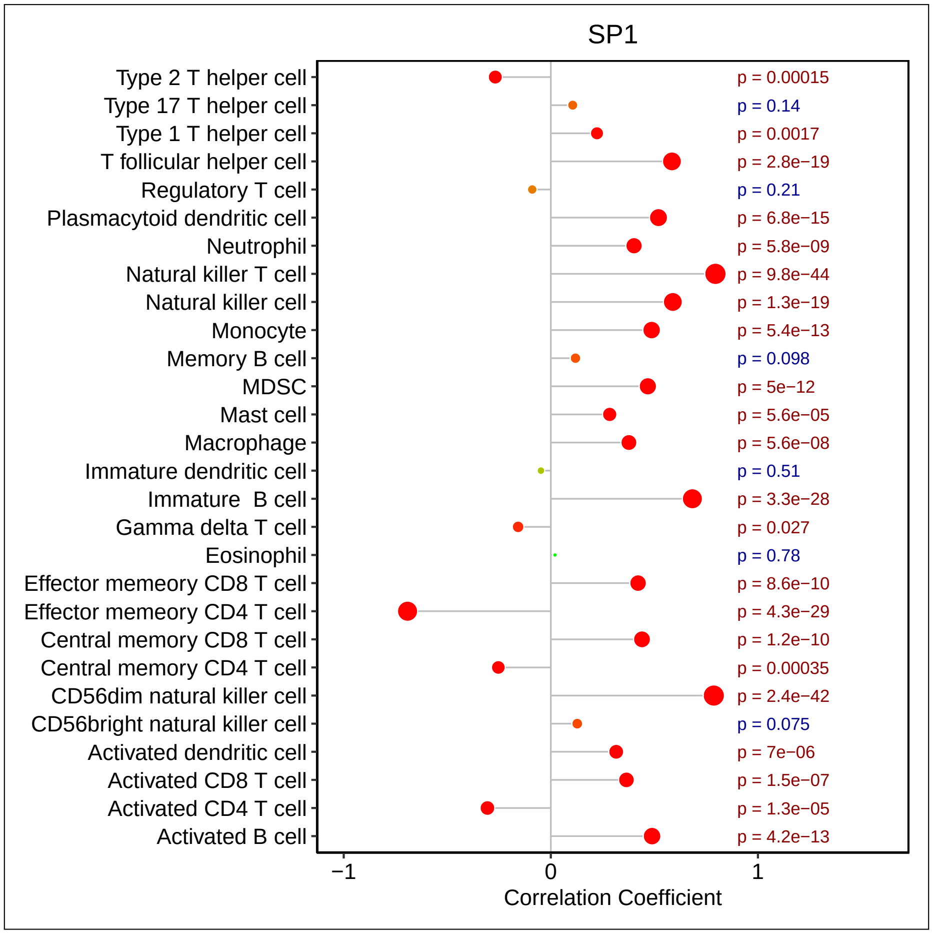

Fig. S3. Immune cell correlation (SP1).

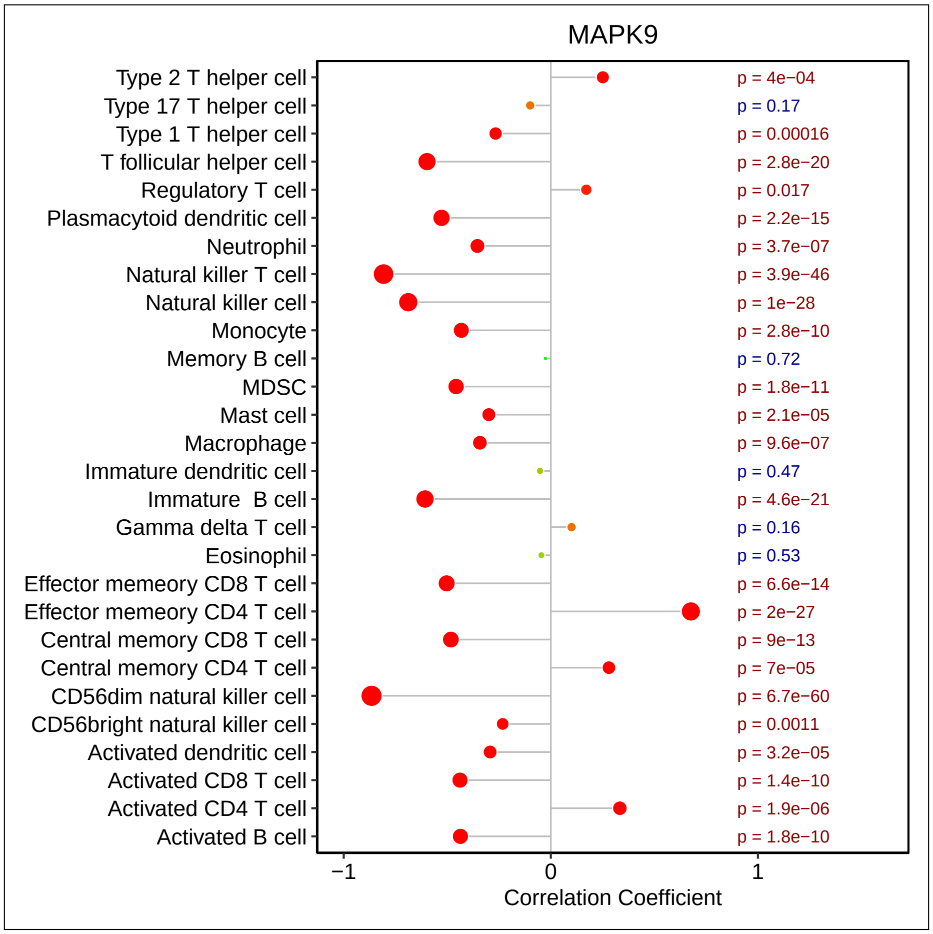

Fig. S4. Immune cell correlation (MAPK9).

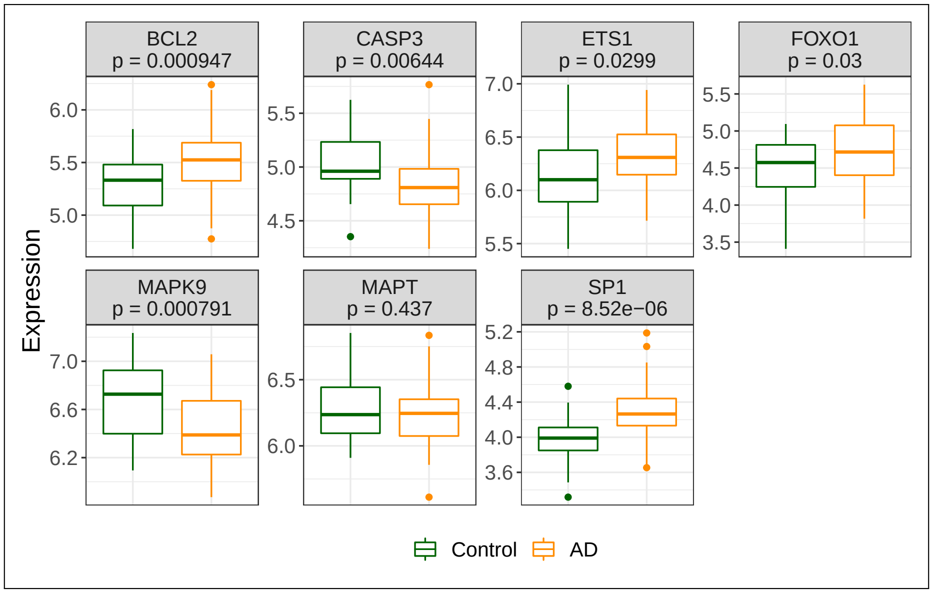

Fig. S5. GSE118553 gene expression.

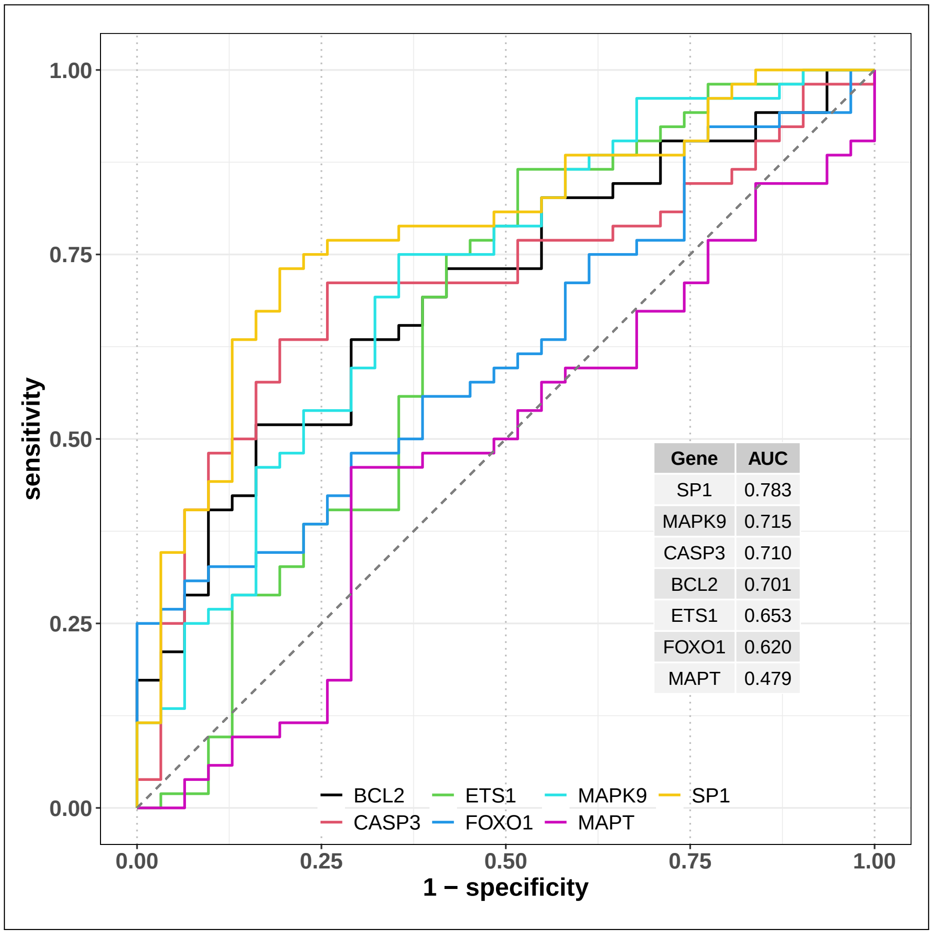

Fig. S6. ROC curve for GSE118553 genes.

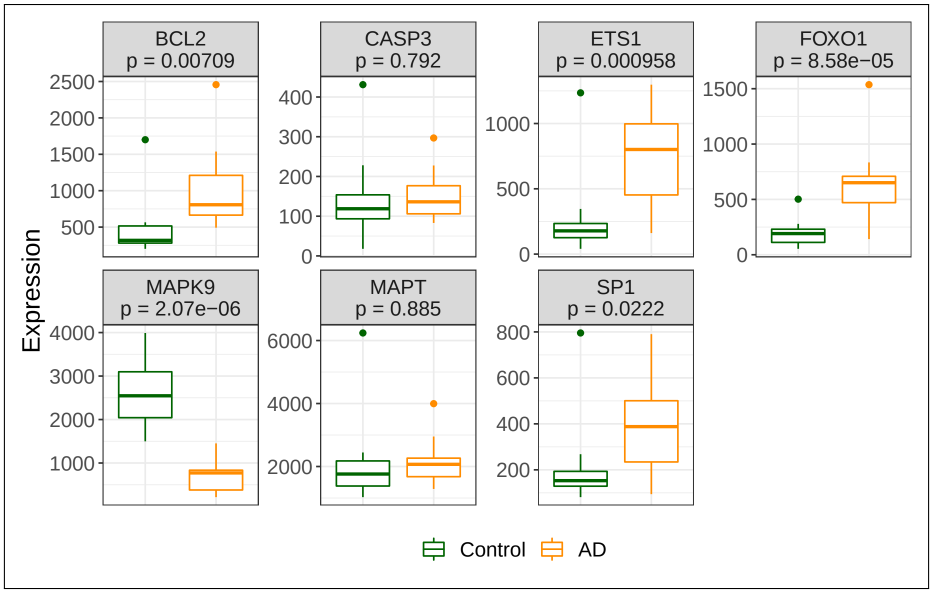

Fig. S7. GSE5281 gene expression.

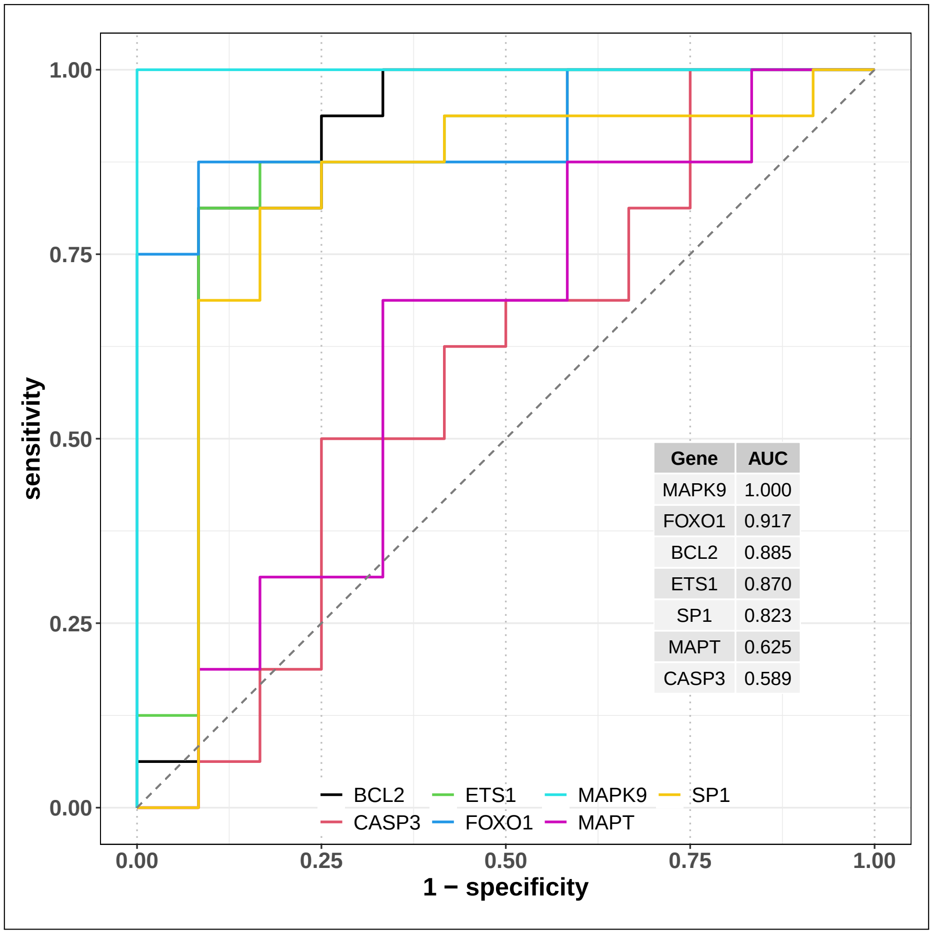

Fig. S8. ROC curve for GSE5281 genes.
